# Infection with Host-Range Mutant Adenovirus 5 Suppresses Innate Immunity and Induces Systemic CD4+ T Cell Activation in Rhesus Macaques

**DOI:** 10.1371/journal.pone.0106004

**Published:** 2014-09-09

**Authors:** Huma Qureshi, Meritxell Genescà, Linda Fritts, Michael B. McChesney, Marjorie Robert-Guroff, Christopher J. Miller

**Affiliations:** 1 Center for Comparative Medicine, University of California Davis, Davis, California, United States of America; 2 California National Primate Research Center, University of California Davis, Davis, California, United States of America; 3 Vaccine Branch, National Cancer Institute, National Institutes of Health, Bethesda, Maryland, United States of America; University of Hawaii Manoa, United States of America

## Abstract

Ad5 is a common cause of respiratory disease and an occasional cause of gastroenteritis and conjunctivitis, and seroconversion before adolescence is common in humans. To gain some insight into how Ad5 infection affects the immune system of rhesus macaques (RM) 18 RM were infected with a host-range mutant Ad5 (Ad5hr) by 3 mucosal inoculations. There was a delay of 2 to 6 weeks after the first inoculation before plasmacytoid dendritic cell (pDC) frequency and function increased in peripheral blood. Primary Ad5hr infection suppressed IFN-γ mRNA expression, but the second Ad5hr exposure induced a rapid increase in IFN-gamma mRNA in peripheral blood mononuclear cells (PBMC). Primary Ad5hr infection suppressed CCL20, TNF and IL-1 mRNA expression in PBMC, and subsequent virus exposures further dampened expression of these pro-inflammatory cytokines. Primary, but not secondary, Ad5hr inoculation increased the frequency of CXCR3+ CD4+ T cells in blood, while secondary, but not primary, Ad5hr infection transiently increased the frequencies of Ki67+, HLADR+ and CD95+/CCR5+ CD4+ T cells in blood. Ad5hr infection induced polyfunctional CD4 and CD8+ T cells specific for the Ad5 hexon protein in all of the animals. Thus, infection with Ad5hr induced a complex pattern of innate and adaptive immunity in RM that included transient systemic CD4+ T cell activation and suppressed innate immunity on re-exposure to the virus. The complex effects of adenovirus infection on the immune system may help to explain the unexpected results of testing Ad5 vector expressing HIV antigens in Ad5 seropositive people.

## Introduction

Since the initial description in the 1950s, adenoviruses have been known as a cause of common childhood respiratory illnesses [1]–[4]. In immunocompetent patients, most of these infections are asymptomatic, mild, or self-limited. The prevalence of Ad5 in North American and other populations has been assessed serologically; almost all subjects tested have Ad5-specific binding antibodies, of which 30–60% also have neutralizing antibody responses [5]–[7]. Adenoviruses infect a broad range of animals in a relatively species-specific manner and adenoviruses can be persistently shed in respiratory secretions and stool [8]–[11]. Adenoviruses isolated from macaque monkey species (rhesus, cynomologus) are in a different phylogenetic group from the human adenoviruses and do not segregate with human Ad5 or other Group C adenoviruses [11].

The biology of wild type adenoviruses is considerably different from adenoviral vectors designed for gene therapy or as vaccine vectors. Adenoviral vectors carry gene deletions to create space for transgenes and/or to attenuate replication, and they are known to induce strong inflammatory responses following administration in humans and animal models [12], [13]. In contrast, infection with wild type Ads suppress host inflammation [14]–[17], a property that may help the viruses establish persistent infection. Adenovirus infections and adenoviral vectors induce neutralizing antibodies and T cell immunity in nonhuman primates (NHP) and humans [10], [18]–[25]. Pre-existing immunity to simian Ads does not affect Ad vector testing in NHP as the immune responses to simian Ads do not cross-reactive with Ad5 [10]. However, very little is known about modulation of innate immunity or immune activation following adenovirus infection in humans or NHP.

Understanding the immune effects of wild type adenovirus infection is of interest because of the prevalence of adenoviruses in humans and the continued development of adenoviruses as gene therapy and vaccine vectors for use in humans [26]. There have been several reports that the immunity to adenoviruses acquired through infection alters the immune response to vaccines in many people [27]–[30]. In the present study, we characterized innate and adaptive immune responses of RM after mucosal infection with a human host range Ad5 mutant (Ad5hr) adapted to replicate in nonhuman primates [31]. We found that Ad5hr infection, after repeated mucosal inoculation, had transient effects on blood plasmacytoid dendritic cell (pDC) frequency and function and altered the mRNA levels of antiviral and pro-inflammatory cytokines in PBMC. Further, Ad5hr infection affected the frequency of Ki67+CD4+, HLADR+ CD4+ and CCR5+ CD4+ T cells and putative CD4+ Treg cells in blood. Finally, Ad5hr infection induced Ad5 hexon-specific T cell responses in blood. Thus, Ad5hr infection of RM affects the host immune system in a dramatic manner that could affect the immune responses to subsequent vaccination with adenoviral vectors.

## Results

### Ad5hr shedding and neutralizing antibody responses in RM

As previously reported [32], 18 male RM were inoculated with Ad5hr orally and intranasally at week 0, then intratracheally at weeks 8 and 12. Ad5 DNA was shed in the nasal secretions of all animals for several days after the first inoculation, and in several animals after the 2nd and 3rd inoculations. By 18 weeks after the first Ad5 hr inoculation, Ad5 neutralizing antibody titers >200 had developed in all but 1 animal [32].

### Delayed increase in number and function of pDC in blood after Ad5hr infection

**Figure 1:**
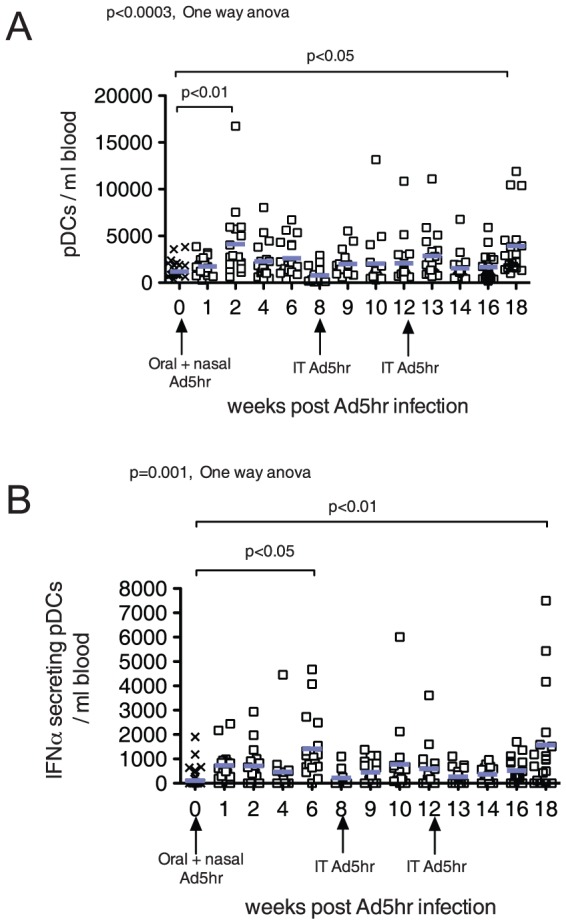
Effect of Ad5hr infection on the number and function of circulating pDC. A) number of pDC per ml of blood. B) number of IFN-α secreting pDCs per ml of blood. Arrows on the X axis indicate the time of Ad5hr inoculation. The mean values for all animals are indicated by blue bars. P values ≤0.05 from the ANOVA and Dunnetts's post-hoc tests are shown.

To assess the effect of Ad5hr infection on pDC (CD3^−^, CD14^−^, CD16^−^, CD19^−^, CD20^−^, CD56^−^, HLA-DR^++^, and CD123^+^) frequency and function, the absolute number of pDC in blood and the ability of blood pDC to produce IFN-alpha in response to in-vitro stimulation with herpes simplex virus were determined [33], [34]. There was a significant increase in the mean number of pDC in blood of the Ad5hr infected RM relative to pre-infection levels at 2 weeks (p<0.01, Fig 1A) and 18 weeks (p<0.05, Fig 1A) after the first Ad5hr inoculation. Relative to pre-infection levels, there was a significant increase in the mean number of IFN-alpha- producing pDCs in the blood of the Ad5hr infected RM 6 weeks (p<0.05, Fig 1B) and 18 weeks after the first Ad5hr inoculation (p<0.01, Fig 1B).

### Increased frequencies of putative regulatory T cells (Tregs) in blood after Ad5hr infection

**Figure 2:**
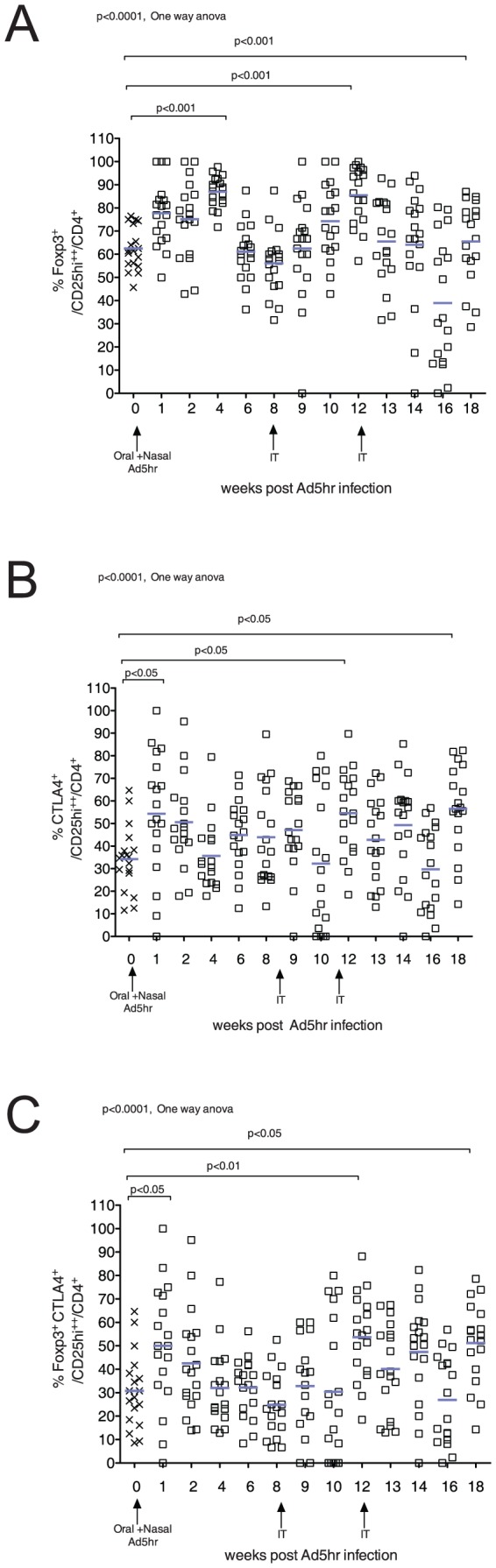
Effect of Ad5hr infection on CD4+ Treg frequency. A) The frequency of FOXP3^+^/CD4^+^ T cells that express a high level of CD25 in blood. B) The frequency of CTLA4^+^/CD25^hi^/ CD4^+^ T cells in blood. C) The frequency of FOXP3^+^/CTLA4+/CD25^hi^/ CD4^+^ T cells in blood. Arrows on the X axis indicate the time of Ad5hr inoculation. The mean values for all animals are indicated by blue bars. P values £ 0.05 from the ANOVA and Dunnetts’s post-hoc tests are shown.

To assess the effect of Ad5hr infection on CD4+ regulatory T cells, we evaluated the frequency of CD4+ Treg (Foxp3+/CD25hi) and a CD4+ Treg subset expressing CTLA4 (Foxp3+/CTLA4+/CD25hi) that is thought to be particularly immunosuppressive, as CTLA4 is required for suppression by Tregs [35]. There was a significant increase in frequency of CD4+ Treg at 4, 12 and 18 weeks (p<0.001, Fig 2A) and CTLA4+/CD4+ Treg frequency at 1, 12 and 18 weeks (p<0.05, Fig 2B) after the first Ad5hr inoculation. After each increase, the frequency of both Treg subsets decreased to the pre-infection levels (Fig. 2).

### Increased frequencies of CCR5+ CD4+ T cells, HLADR+ CD4+ T cells and Ki67+ CD4+T cells in blood after the second Ad5hr re-infection

**Figure 3:**
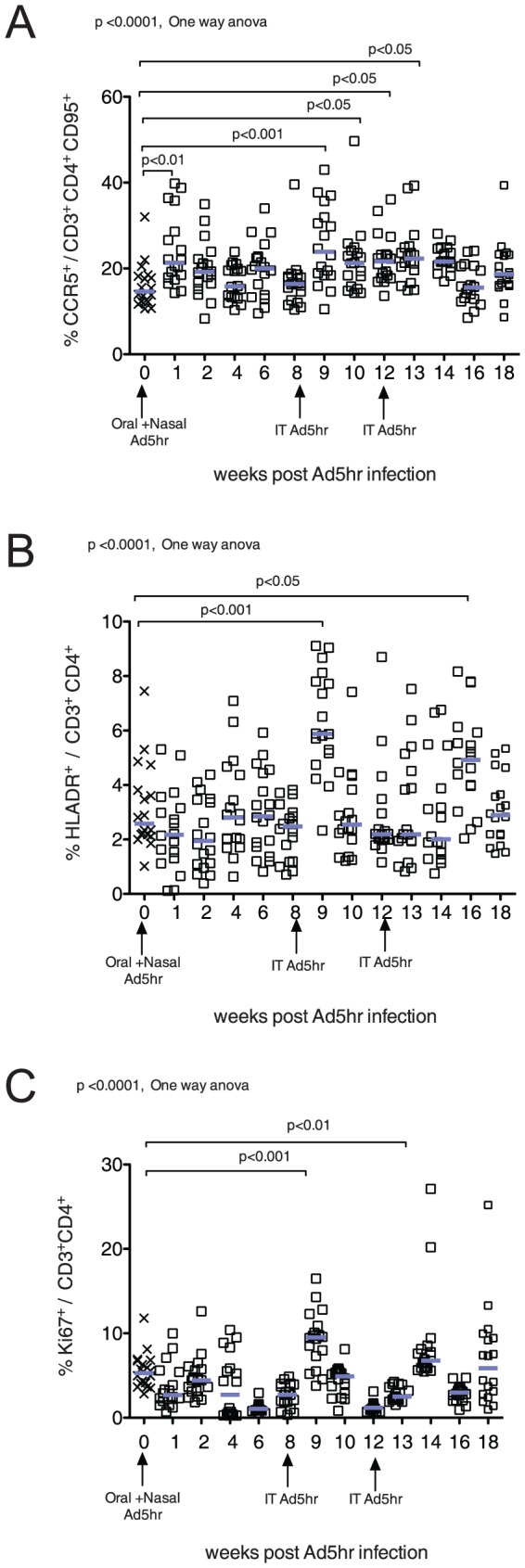
Effect of Ad5hr infection on the frequency of circulating CD4+T cells expressing activation markers. A) The percentage of CD95+/CD3+/CD4+ cells expressing CCR5. B) The percentage of CD3+/CD4+ cells expressing a high level of HLA-DR. C) The percentage of CD3+/CD4+ cells expressing Ki67. Arrows on the X axis indicate the time of Ad5hr inoculation. The mean values for all animals are indicated by blue bars. P values ≤0.05 from the ANOVA and Dunnetts's post-hoc tests are shown.

The frequency of CCR5+ CD4+ T memory cells in blood increased at week 1 after primary Ad5hr infection (p<0.01, Fig 3A), then returned to pre-infection levels. However, after the second and third Ad5hr inoculations at weeks 8 and 12, the frequency of circulating CCR5+ CD4+ T cells at weeks 9, 10, 12 and 13 (p<0.001–0.05, Figure 3A) were elevated. After the second and third Ad5hr inoculations, there were also significant increases in the frequency of HLADR+CD4+ T cells at week 9 (p<0.001, Fig 3B) and week 16 (p<0.05, Fig 3B). We also found that the frequency of activated/proliferating Ki67+ CD4+ T cells was increased at week 9 (p<0.001, Fig 3C) and week 14 (p<0.01, Fig3C), after the second and third Ad5hr inoculations.

### Reduced expression of proinflammatory cytokine and chemokine mRNAs in PBMC after Ad5hr infection

**Figure 4:**
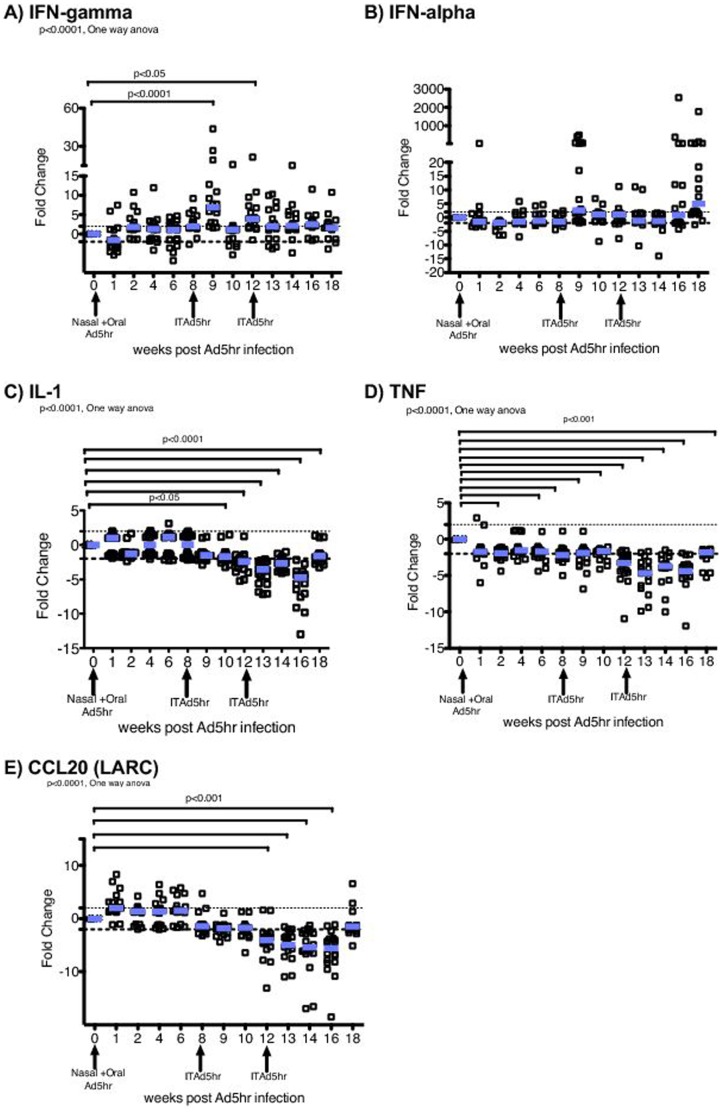
Effect of Ad5hr infection on proinflammatory cytokine and chemokine gene expression in PBMC. A) The fold-change of IFN-gammamRNA in PBMCs; B) IFN-α; C) IL-1; D) TNF and E) CCL20. The fold-change in target gene mRNA levels in study animals post-Ad5hr infection relative to the same target gene mRNA level before Ad5hr infection was calculated as described in Materials and Methods. Arrows on the X axis indicate the time of Ad5hr inoculation. The mean values for all animals are indicated by blue bars P values ≤0.05 from the ANOVA and Dunnetts's post-hoc tests are shown.

The mRNA levels of IFN-gamma, IFN-alpha, IL-1, CCL20 and TNF in PBMC were variably affected by the primary Ad5hr infection (Fig 4 A–E), but IL-1, CCL20 and TNF were significantly decreased after subsequent Ad5 exposures (Fig 4). In contrast, there were small but significant increases in IFN-gamma mRNA expression in PBMC (p<0.05, Fig 4 A) after the second Ad5hr exposure (weeks 9 & 12), which decreased following the 3rd exposure.

### Ad5hr infection induced polyfunctional hexon-specific T cell responses in blood

**Figure 5:**
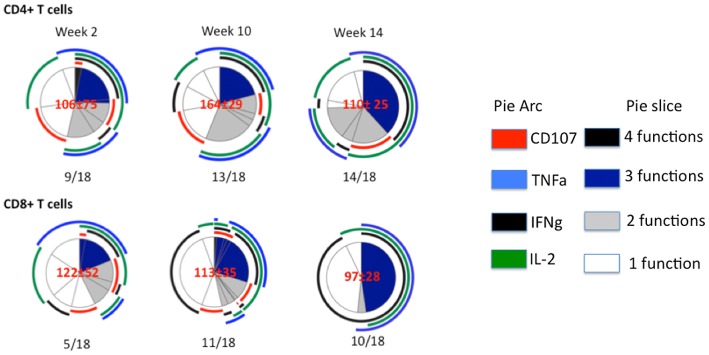
Ad5 hexon-specific T cell responses in PBMCs after Ad5hr infection as detected by multiparameter flow cytometry. Pie charts present the average CD4+ or CD8+T cell responses for all positive animals at weeks 2, 10 and 14 from the first Ad5hr inoculation, as described in Materials and Methods. Pie slice colors denote the number of positive functions (expression of IL-2, IFN-γ, TNF and CD107 degranulation). Pie arc colors denote the 4 functions separately. Red numbers in the center of pie charts are the mean ± standard deviation of hexon-specific T cells normalized to 10^5^ CD3+ T cells. Fractional numbers below a pie chart refer to the number of animals in a group of 18 that responded.

We evaluated the T cell responses in PBMCs of the Ad5hr - inoculated RM to 3 pools of overlapping peptides representing the aa sequence of the Ad5 hexon protein. Moderate Ad5 hexon-specific CD8+ and CD4+ T cell responses (>2 fold increase vs pre-infection) were detected in about half of the RM at week 2 (Table 1). The hexon-specific T cells induced after primary Ad5hr infection produced 1–3 of the cytokines measured (Fig 5). Subsequent Ad5hr exposures (week 8 and 12) led to detectable hexon-specific CD4+ T cell responses in about 77% of RM (Table 1). By week 14, after 3 mucosal Ad5hr exposures, the hexon-specific CD4+ and CD8+T cell responses were more polyfunctional than the hexon-specific T cells detected at week 2 (Fig 5). Of note, the breadth of the hexon-specific T cell responses, as measured by the number of peptide pools eliciting positive responses, was maximum at week 10 after the second Ad5hr inoculation and most restricted at week 14 after 3 Ad5hr inoculations (Table 1).

**Table 1:** Adenovirus hexon-specific T cell responses in PBMCs after Ad5hr infection.

	Week 2		Week 10		Week 14	
Peptides/T cell	# events*	% positive†	# events	% positive	# events	% positive
Pool 1/CD4	101±36	44	175±41‡	78	68±19	56
Pool 2/CD4	92±30	44	264±48‡	72	65±16	56
Pool 3/CD4	106±75‡	50	164±29‡	72	110±25‡	78
Pool 1/CD8	186±76‡	33	85±14	67	73±18	28
Pool 2/CD8	73±34	44	99±14	61	43±12	56
Pool 3/CD8	122±52‡	28	113±35‡	61	97±28‡	56

* Flow cytometric events (mean ±SE) normalized to 10^5^ CD3^+^ T cells.

† Percentage of responders /18 animals tested.

‡ Responses that were ≥ two-fold compared to pre-infection levels were considered strongly positive.

## Discussion

We previously showed that after RM are inoculated with Ad5hr, virus is consistently shed in feces and respiratory secretions after the first Ad5hr inoculation but shedding is intermittent after the second and third inoculations [32]. The pattern of virus shedding in Ad5hr-infected RM mirrors Ad5hr shedding in humans. Adenovirus shedding from the lower GI tract is common in children during symptomatic and asymptomatic infections and it also occurs in healthy adults [36]–[39]. A recent study found adenoviruses in 7.1% of approximately12,000 respiratory samples from pediatric populations with respiratory symptoms, and there was a high rate of adenovirus co-infection (21.7%) with multiple serotypes in many positive samples [40]. Although Ad5 infected RM and humans develop potent neutralizing antibody responses and Ad5-specific T cells responses [10], [18]–[25], [32], the pattern of virus shedding suggests that this immunity does not prevent re-infection. Further anti-Ad5 immunity is often incapable of completely clearing Ad5 infections as the virus can persist in mucosal surfaces of humans [8] and RM [9] for months.

The tropism of Ad5hr in RM and Ad5 in humans is similar and could explain how Ad5 infection affects innate immunity. The coxsakie virus adenovirus receptor (CAR) which binds to the knob domain of the fiber protein, is described as the primary receptor for human adenoviruses [41], [42]. However, a number of cells that do not express CAR support adenovirus replication. In fact CAR has been shown to play a minor role in Ad5 infection of many cell types including epithelial cells. Further, human monocytes and dendritic cells (DCs) are susceptible to Ad5 infection despite their lack of CAR expression [43], [44]. Although Langerhans cells and dermal DCs from skin express CAR, blocking CAR does not block Ad5 infection, indicating that other receptor pathways mediate viral entry into these cells [45]. Ad5hr in rhesus macaques and Ad5 in humans target lung and gut macrophages and dendritic cells [9]. Thus biology of Ad5hr infection of RM and Ad5 infection of humans are very similar. The affinity of Ad5 for long-lived antigen presenting cells, macrophages and DC in mucosal sites may partially explain the ability of these infections to suppress innate immunity and induce systemic CD4+ T cell activation.

Here we find report that Ad5hr infection affects the RM immune system in a variety of ways, with repeated exposures having complex and additive effects. The host innate immune system was suppressed by Ad5hr infection as evidenced by changes in the number and function of pDC in blood and decreased expression of pro-inflammatory and immunoregulatory cytokines and chemokine mRNAs in PBMC. Ad5hr infection also altered the adaptive immune system of the host, transiently increasing the frequency of activated T cells and CD4+ T cells expressing Treg markers in blood.

There was a transient increase in pDC frequency and function 2 weeks after the initial Ad5hr exposure (Fig 1A& 1B); however, there was no change from pre-inoculation levels in the pDC frequency or function at week 1 after Ad5hr inoculation while viral replication was at its peak. Thus primary Ad5hr exposure did not appear to induce strong antiviral pDC immune responses in blood. To further understand the Ad5hr induced effects on the immune system, we characterized the antiviral/proinflammatory cytokine and chemokine mRNA expression levels in PBMCs of Ad5hr-infected RM. There was little change in cytokine or chemokine mRNA levels in PBMC at week 1 after the first Ad5hr exposure. Thus, at the gene expression level primary Ad5hr exposure did not induce, and may have suppressed, strong antiviral and pro-inflammatory immune responses in blood 7 days after oral inoculation. In contrast, 7- 14 days after subsequent Ad5hr exposures there was a very transient increase in IFN-gamma mRNA in PBMCs. Although IFN-gamma is a key molecule of antiviral defenses [46], [47], it also increases the levels of immune activation and inflammation in response to infection [48], [49]. Thus the expression of molecules associated with innate antiviral response was suppressed in PBMC by primary Ad5 infection and, with the exception of IFN-gamma, secondary Ad5 infections/exposures reinforced and broadened this effect.

Human pDC infected *in vitro* with Ad5 or other Group C adenoviral strains do not produce inflammatory cytokines, in contrast to Group B and E strains [10], and in marked contrast to E1-deleted Ad5 vectors [50]. It seems likely that the inhibition of pDC responses and antiviral/proinflammatory cytokine and chemokine responses that we found in PBMCs of RM infected with Ad5hr is due to the expression of immunomodulatory viral gene products in the initial stages of infection when viral replication is unrestricted by host immunity. The viral proteins, E1 & E3, are the most likely mediators of this immunomodulation. These viral proteins suppress NFκB activation and the subsequent inflammatory response following Ad5 infection [14]–[16]. However, after the second and third Ad5hr inoculations, Ad5hr-specific immune responses may have blunted virus replication, with reduced expression of E1 & E3 and less suppression of interferon but not other innate immune responses.

After primary Ad5hr infection there was an increased frequency of activated CD4+ T cells in blood, although the levels of these cells quickly returned to baseline. However, subsequent Ad5hr exposures induced recurrent CD4+T cell activation, including expression of Ki67, a marker associated with T cell proliferation. Thus, as with IFN-gamma expression in PBMC, repeated Ad5hr exposure was necessary to induce systemic CD4+ T cell activation, which although not sustained, recurred upon re-exposure to Ad5. Similar transient CD4+ T cell activation was observed in RM after vaccination with an E1-deleted, replication defective Ad 5 vector [51]. As people are likely to be repeatedly exposed to Ad5, frequent periods of transient T cell activation probably occur with some frequency in Ad5 seropositive individuals.

A recent report showed that Ad5 immune complexes interact with Fc receptors on DC to enter the endosomal compartment where Ad5 genomic DNA interacts with TLR9 [52]. This TLR9 ligation activates DCs and results in production of proinflammatory cytokines. If this type of interaction occurs *in vivo*, it could activate Ad5-specifc and bystander T cells after secondary Ad5hr exposures. Because as we previously reported Ad5hr infection of RM induces Ad5-specific antibodies [32], this phenomenon could explain the enhanced virus transmission after Ad5 seropositive individuals were immunized with rAd5 Merck Step vaccine.

The frequency of Tregs in blood after the primary Ad5hr infection increased (Fig 2). This response to Ad5hr infection by RM is not unique, as upregulation of Tregs early after pathogen infection [53]–[55] with concomitant modulation of pathogen-specific immunity has been recently reported [54], [56]. Furthermore, CTLA4 expression on Tregs is associated with Treg-mediated immune suppression 36 [57]. It has been shown that Tregs promote pathogen persistence in leishmania and TB infection [53], [55].

After Ad5hr infection RM develop weak and variable hexon-specific CD4+ and CD8+ T cell responses in the blood. These hexon-specific T cells predominantly secreted 1–3 cytokines in various combinations (Table 1, Fig 5). Secondary Ad5hr exposures expanded hexon-specific CD4+ T cell responses (Table 1) with increases in polyfunctional T cells; especially IFN-gamma +/TNF+/IL-2+ CD4+ T cells (Fig 5). Similarly Ad5-specific CD4+ and CD8+ T cells are found in humans and CD4+ T cell responses are focused on the hexon protein [21], [23].

Although, preexisting Ad5 specific immunity does not prevent infection, it does affect the immunogenicity of Ad5-based vaccines in RM and humans [27]–[30], [32]. Adenovirus Immunity at the time of immunization with the Step vaccine modified T helper cell cytokine responses to the vaccine [28], reduces innate immune responses [30] and is associated with relatively poor HIV-specific T cell responses in both human and RM Step vaccine recipients [27], [32]. Ad5-immunity present in the host prior to Ad5 vaccine immunization result in weaker HIV or SIV-specific T cell responses in that less vaccinees respond, and the magnitude, antigenic breadth and cytokine functions of the HIV/SIV-specific T cells were reduced compared to their Ad5 seronegative counterparts [27], [32]. How the pre-existing Ad5 immunity affects the responses to vaccines has not been rigorously determined. The lower immune responses to HIV antigens in Ad5 seropositive people immunized with an Ad5 vector is assumed to be due to the killing of these APC as they express HIV and Ad5 antigens by pre-existing Ad5-specific memory cytotoxic T cells. In addition, preexisting Ad5-specific antibodies could form immune complexes with Ad5 vaccine virus that activate and expand Ad5-specific memory T cell responses. These Ad5-specific T cells, in turn, could limit HIV-specific immune responses to the vaccine by killing the DCs co-expressing Ad5 and HIV antigens [52].

The results reported here further demonstrate that Ad5hr infection alters the host immune system in complex ways that could affect the host response to subsequent vaccination, particularly if an immunization was given just before, or after, a secondary Ad5 infection/exposure. The Step Trial HIV vaccine resulted in enhanced infection in some Ad5-seropositive, uncircumcised vaccinees [58]. A vaccine study in macaques that modeled the Step Trial recapitulated the lack of protection and a greater risk of infection in immunized macaques with pre-existing Ad5 seropositivity [32]. In both the Step trial and the monkey study, enhanced susceptibility to infection was only seen in individuals with a prior or ongoing Ad5 infection. However, there is no evidence from these studies, or from HVTN 505 (the most recent efficacy trial of a HIV vaccine using a replication defective Ad5 vector in Ad5 seronegative men), that a replication defective Ad5 vector vaccine can enhance HIV transmission in individuals that are not, or have not been, infected with Ad5. Further, Ad5 infection alone is not associated with increased risk of HIV infection AIDS [59]. Thus the results reported here, taken together with the results of the clinical trials, raise the possibility that Ad5 infection alters the responses to immunizations in general. Additional experiments are needed to determine the relative contributions of the Ad5 vector and Ad5 infections to the unexpected outcomes of the Step trial and nonhuman primate studies.

## Materials and Methods

### Ethics Statement

As previously reported [32], the captive-bred 4–9 year old male rhesus macaques (Macaca mulatta) used in this study were from the California National Primate Research Center and they were housed in accordance with the recommendations of the Association for Assessment and Accreditation of Laboratory Animal Care International Standards and with the recommendations in the Guide for the Care and Use of Laboratory Animals of the National Institutes of Health. The Institutional Animal Use and Care Committee of the University of California, Davis, approved these experiments (Protocol # 15835). When immobilization was necessary, the animals were injected intramuscularly with 10 mg/kg of ketamine HCl (Parke-Davis, Morris Plains N.J.). All efforts were made to minimize suffering. Details of animal welfare and steps taken to ameliorate suffering were in accordance with the recommendations of the Weatherall report, “The use of non-human primates in research”. Animals were housed in an air-conditioned facility with an ambient temperature of 21–25°C, a relative humidity of 40%–60% and a 12 h light/dark cycle. Animals were individually housed in suspended stainless steel wire-bottomed cages and provided with a commercial primate diet. Fresh fruit was provided once daily and water was freely available at all times. A variety of environmental enrichment strategies were employed including housing of animals in pairs, providing toys to manipulate and playing entertainment videos in the animal rooms. In addition, the animals were observed twice daily and any signs of disease or discomfort were reported to the veterinary staff for evaluation. For sample collection, animals were anesthetized with 10 mg/kg ketamine HCl (Park-Davis, Morris Plains, NJ, USA) or 0.7 mg/kg tiletamine HCl and zolazepan (Telazol, Fort Dodge Animal Health, Fort Dodge, IA) injected intramuscularly. The animals were sacrificed by intravenous administration of barbiturates prior to the onset of any clinical signs of disease.

### Ad5hr infection

As previously reported [32], 18 RM were infected with 1.5×10^9^ infectious particles/dose/route of Ad5hr [31], by nasal & oral routes at week 0 and by the intratracheal route at week 8 and 12.

### Isolation of lymphocytes from blood

PBMCs were isolated from heparinized blood using Lymphocyte Separation Medium (ICN Biomedicals). PBMC samples were frozen in 10% dimethyl sulfoxide (DMSO; Sigma-Aldrich) and 90% fetal bovine serum (Gemini BioProducts), stored in liquid nitrogen until future use in immunological assays [32].

### T cell phenotyping in blood

Blood samples were surface stained with 4 color panels of antibodies. Panel 1; anti-CD3 PerCP clone no. SP34, anti-CD4 APC clone no. M-T477, anti-CD95 FITC clone no. DX2 and anti-CCR5 PE clone no. 3A9. Panel 2; anti-CD3 PerCP clone no. SP34, anti-CD8 APC clone no. SK1, anti-CD38 PE clone OKT10, anti-HLADR FITC clone no G46-6. Surface stained samples were fixed with Q prep (Beckman Coulter). All antibodies were purchased from Pharmingen/Becton-Dickinson, San Diego, CA, unless specified. Data were acquired using a FACS Calibur cytometer (Becton Dickinson), and analyzed using FlowJo software (Treestar, Inc.) and a Macintosh G5 computer (Apple, Inc.). At least 100,000 small lymphocyte events were collected from each tube analyzed.

### Intracellular staining for Ki-67 in blood

Blood samples were surfaced stained with the following antibodies; anti-CD3-Pacific Blue (Clone SP3F2), anti-CD4-Amcyan (Clone L200), anti-CD8-Cy7APC (Clone SK1) and anti-CXCR3-PE (clone 1C -6). Thereafter, surface stained samples were washed and permeabilized with 0.5% saponin for intracellular staining, and then stained with Ki67-FITC (Clone B56) for 20 min. Ki67- stained samples were washed with the permeabilizing buffer and fixed with 1% paraformaldehyde.

### Intracellular staining for cytokine and degranulation markers

As previously described in detail [32], for intracellular staining of PBMCs, cryopreserved samples were thawed and rested overnight at 37°C, in 5% CO2 atmosphere, in RPMI media (Gibco, Invitrogen Inc.) containing 10% fetal calf serum. To stimulate cells, 3 peptide pools spanning the hexon protein, 20mer peptides overlapping by 10 residues (Anaspec, Inc.), were prepared at 5 µg/ml/peptide in DMSO. The negative control contained co-stimulatory molecules and DMSO, and the positive control was staphylococcal enterotoxin B (0.2 µg/ml, Sigma-Aldrich). At least 100,000 events in the forward scatter/side scatter lymphocyte gate were acquired. Further, samples that had a large discrepancy between the number of events in the negative control and the peptide-stimulated tubes were eliminated. The background level of cytokine staining varied from sample to sample. Samples were considered positive in which, after subtracting the negative control, there were at least 5 positive events for a single functional marker, 3 positive events for two or more functional markers, and the sum of the different combinations of responses represented at least 10 events. In addition, a sample was not considered positive for a particular combination of functions if the frequency of T cells responding with that particular combination of functions was lower than 0.02%. The software program Simplified Presentation of Incredibly Complex Evaluations (SPICE; a gift from M. Roederer, Vaccine Research Center, NIAID/NIH) was used to create the pie charts that represent the average of all positive responses [60].

### Relative quantification of cytokine/ chemokine mRNA expression levels in PBMC

The mRNA levels were determined by real-time PCR as described previously [61], [62] for IL-1, IFN-alpha, IFN-gamma, TNF and CCL20. The comparative threshold cycle (Ct) method was used for quantification of mRNA levels (User Bulletin No. 2, ABI PRISM 7700 Sequence Detection System, Applied Biosystems). GAPDH was used as the reference gene and all samples were tested in duplicate. A ΔCt value was generated by subtracting the Ct value of GAPDH from the Ct value of the target mRNA. To compare the target gene mRNA levels pre- and post- Ad5hr infection, the mean ΔCt of a target gene at a pre-infection time point in an individual animal was used as a reference value to generate the ΔΔCt for the same target gene in the that particular animal at post-infection time points [63]. Thus, the ΔΔCt value of a target gene in study animals at post-infection time point is equal to difference between ΔCt value of the target gene in the study animal at a post-infection time point and the ΔCt value of the target gene in that animal at the pre-infection time point. The fold-change in target gene mRNA levels in study animals post-Ad5hr infection relative to target gene mRNA levels in the study animals pre-Ad5hr infection was calculated using one of 2 formulas based on the ΔΔCt:

1. Fold change of decreased mean mRNA level = −1/2^−ΔΔCt^ (If the ΔΔCt was positive).
2. Fold change of increased mean mRNA level  = 2^−ΔΔCt^ (If the ΔΔCt was negative).

### pDC enumeration and assessment of IFN-alpha production

Plasmacytoid DCs were phenotyped using a lineage marker Ab cocktail (CD3, CD14, CD16, CD19, CD20, CD56) and anti-CD123, as previously described [34].

### Statistical analyses

Data are reported as the median and the standard error of the mean (SEM) for each animal group using Prism 5.0 software (GraphPad Software). Statistical analyses were performed by one-way ANOVA with Dunnetts's multiple comparison test if more than two groups were compared. The flow cytometric data analysis program, SPICE, was used to analyze T cell responses detected by polychromatic flow cytometry. A P value of <0.05 was considered significant.
